# Supplementary material for: Insights from a Pan India Sero-Epidemiological survey (Phenome-India Cohort) for SARS-CoV2
Source: eLife. 2021 Apr 20;10:e66537. doi: 10.7554/eLife.66537 (PMC8118652; doi:10.7554/eLife.66537)
Supplement: Figure 1—source data 1. — Lab, district, and date of collection (DOC) (columns A–C), total samples collected, number of seropositive samples, seropositivity in percentage (rounded off), and number of samples tested and found positive for neutralizing antibody (NAB). Columns (E–I) Number of confirmed cases and tests done for respective states 15 days and prior and after the DOC. Columns (L–N) Data obtained from http://www.covid19India.org. State data has been utilized as a surrogate to city/district data for city/district data was not available for number of cases/tests done for many. [file elife-66537-fig1-data1.docx]

| A | B | C |
| --- | --- | --- |
| **Lab/Institute** | **District/State** | **Date of Collection** |
| CSIR-Advanced Materials and Processes Research Institute (CSIR-AMPRI), Bhopal | Bhopal | 15th Sep |
| CSIR-Central Building Research Institute(CSIR-CBRI), Roorkee | Haridwar | 15th Sep |
| CSIR-Centre for Cellular Molecular Biology(CSIR-CCMB), Hyderabad | Hyderabad | 10th Sep |
| CSIR-Central Drug Research Institute(CSIR-CDRI), Lucknow | Lucknow | 09th Sep |
| CSIR-Central Electrochemical Research Institute(CSIR-CECRI), Karaikudi | Sivaganga | 17th Sep |
| CSIR-Central Electronics Engineering Research Institute(CSIR-CEERI), Pilani | Jhunjunu | 04th Sep |
| CSIR-Central Food Technological Research Institute(CSIR-CFTRI), Mysore | Mysuru | 15th Sep |
| CSIR-Central Glass Ceramic Research Institute(CSIR-CGCRI), Kolkata | Kolkata | 26th Aug |
| CSIR-Central Institute of Medicinal Aromatic Plants(CSIR-CIMAP), Lucknow | Lucknow | 23rd Sep |
| CSIR-Central Institute of Mining and Fuel Research(CSIR-CIMFR) Dhanbad | Dhanbad | 15th Sep |
| CSIR-Central Leather Research Institute(CSIR-CLRI), Chennai | Chennai | 17th Sep |
| CSIR-Central Road Research Institute(CSIR-CRRI), New Delhi | South Delhi | 22nd July |
| CSIR-Central Scientific Instruments Organisation(CSIR-CSIO), Chandigarh | Chandigarh | 18th Sep |
| CSIR-Central Salt Marine Chemicals Research Institute(CSIR-CSMCRI), Bhavnagar | Bhavnagar | 17th Sep |
| CSIR-National Aerospace Laboratories(CSIR-NAL), Bengaluru and 4 PI* | Bengaluru | 08th Sep |
| CSIR-Institute of Genomics and Integrative Biology(CSIR-IGIB), Delhi* | South Delhi | 17th June |
| CSIR-Institute of Himalayan Bioresource Technology(CSIR-IHBT), Palampur | Kangra | 11th Sep |
| CSIR-Indian Institute of Chemical Biology(CSIR-IICB), Kolkata | Kolkata | 25th Aug |
| CSIR-Indian Institute of Chemical Technology(CSIR-IICT), Hyderabad | Hyderabad | 26th July |
| CSIR-Indian Institute of Integrative Medicine(CSIR-IIIM), UT of J&K | Jammu | 09th Sep |
| CSIR-Indian Institute of Petroleum(CSIR-IIP), Dehradun | Dehradun | 26th Aug |
| CSIR-Institute of Minerals and Materials Technology(CSIR-IMMT), Bhubaneswar | Khordha | 25th Aug |
| CSIR-Institute of Microbial Technology(CSIR-IMTECH), Chandigarh | Chandigarh | 21st Sep |
| CSIR-National Chemical Laboratory(CSIR-NCL), Pune | Pune | 12th Sep |
| CSIR-National Environmental Engineering Research Institute(CSIR-NEERI), Nagpur | Nagpur | 20th Aug |
| CSIR-North - East Institute of Science and Technology(CSIR-NEIST), Jorhat | Jorhat | 20th Aug |
| CSIR-National Geophysical Research Institute(CSIR-NGRI), Hyderabad | Hyderabad | 17th Sep |
| CSIR-National Institute For Interdisciplinary Science and Technology(CSIR-NIIST),Thiruvananthapuram | Thiruvananthapuram | 04th Sep |
| CSIR-National Institute of Oceanography(CSIR-NIO), Goa | North Goa | 22nd Sep |
| CSIR-National Institute of Science, Technology And Development Studies(CSIR-NISTADS), New Delhi | Central Delhi | 09th Sep |
| CSIR-National Physical Laboratory(CSIR-NPL), New Delhi | Central Delhi | 1st Sep |
| CSIR-UNIT : Traditional Knowledge Digital Library(CSIR-TKDL), New Delhi | South West Delhi | 27th Aug |
| CSIR-UNIT : Human Resource Development Centre(CSIR-HRDC), Ghaziabad | Ghaziabad | 29th July |
| Head Quarters | Central Delhi | 21st Aug |
| Pusa Complex | Central Delhi | 16th Sep |
| Maharani Bagh Apartment | South Delhi | 08th Aug |
| NEERI_Delhi Unit | South West Delhi | 31st Aug |
| NEERI_Hyd Unit | Hyderabad | 10th Sep |
| *For IGIB, New Delhi and NAL, Bengaluru , the samples collection was spread over 2-3 weeks. |  |  |

Figure 1 and Figure 2B Source Data: Data for all labs/centers utilized for Figure 2A and Figure 2B. Lab, District and Date of Collection (DOC) [Column A-C] Total Samples Collected, Number of sero-positive samples, sero-positivity in percentage (rounded Off), Number of samples tested and found positive for NAb. Columns [E-I] Number of confirmed cases and tests done for respective states 15 days and prior and after the DOC. Columns [L-N] Data obtained from [www.covid19India.org](http://www.covid19India.org)) (State Data has been utilized as a surrogate to City/ District Data for City/District data was not available for number of cases/tests done for many).

D E F G H I

| **Lab/Institute** | **Total**  **Samples** | **Positive**  **Samples** | **Sero-Positivity**  **(%)** | **Samples Tested**  **for NAb** | **Samples Positive**  **for NAb** |
| --- | --- | --- | --- | --- | --- |
| **AMPRI** | 195 | 12 | 6 | 12 | 11 |
| **CBRI** | 122 | 9 | 7 | Nil | Nil |
| **CCMB** | 407 | 118 | 29 | 118 | 116 |
| **CDRI** | 287 | 12 | 4 | 12 | 11 |
| **CECRI** | 167 | 19 | 11 | 19 | 19 |
| **CEERI** | 382 | 6 | 2 | 6 | 3 |
| **CFTRI** | 294 | 29 | 10 | 29 | 26 |
| **CGCRI** | 98 | 12 | 12 | 12 | 12 |
| **CIMAP** | 165 | 16 | 10 | 16 | 16 |
| **CIMFR** | 269 | 31 | 12 | 31 | 26 |
| **CLRI** | 437 | 69 | 16 | 65 | 63 |
| **CRRI** | 145 | 17 | 12 | 17 | 15 |
| **CSIO** | 223 | 14 | 6 | 14 | 13 |
| **CSMCRI** | 94 | 12 | 13 | 12 | 11 |
| **NAL & 4PI** | 639 | 61 | 10 | 61 | 59 |
| **IGIB** | 395 | 53 | 13 | 53 | 50 |
| **IHBT** | 471 | 9 | 2 | 9 | 8 |
| **IICB** | 288 | 28 | 10 | 28 | 28 |
| **IICT** | 1027 | 133 | 13 | 119 | 117 |
| **IIM** | 126 | 6 | 5 | 6 | 6 |
| **IIP** | 319 | 4 | 1 | Nil | Nil |
| **IMMT** | 398 | 4 | 1 | 4 | 2 |
| **IMTECH** | 232 | 22 | 9 | 22 | 22 |
| **NCL** | 367 | 25 | 7 | 25 | 25 |
| **NEERI** | 96 | 0 | 0 | 0 | 0 |
| **NEIST** | 708 | 67 | 9 | 67 | 66 |
| **NGRI** | 585 | 111 | 19 | 109 | 107 |
| **NIIST** | 368 | 3 | 1 | 3 | 2 |
| **NIO** | 138 | 6 | 4 | 6 | 6 |
| **NISTADS** | 140 | 40 | 29 | 40 | 36 |
| **NPL** | 245 | 21 | 9 | 21 | 19 |
| **TKDL** | 91 | 11 | 12 | 11 | 10 |
| **HRDC** | 72 | 9 | 13 | 9 | 9 |
| **HQ** | 109 | 18 | 17 | 18 | 17 |
| **Pusa Comp.** | 99 | 22 | 22 | 22 | 22 |
| **Apartments** | 119 | 12 | 10 | 12 | 8 |
| **NEERI,DEL** | 56 | 8 | 14 | 8 | 6 |
| **NEERI,HYD** | 54 | 9 | 17 | 9 | 9 |

| K | L | M | M | N |
| --- | --- | --- | --- | --- |
| **Lab/Institute** | **No. of cases 15 days before DOC** | **No. of Tests 15 days before DOC** | **Number of cases 15 days after DOC** | **No. of Tests 15 days after DOC** |
| **AMPRI** | 63965 | 1377427 | 128047 | 2035134 |
| **CBRI** | 19827 | 394015 | 49000 | 712124 |
| **CCMB** | 111688 | 1082094 | 181627 | 2741836 |
| **CDRI** | 197388 | 4796488 | 374277 | 9145828 |
| **CECRI** | 439959 | 4964141 | 608885 | 7526688 |
| **CEERI** | 66619 | 2033646 | 113124 | 2826262 |
| **CFTRI** | 342423 | 2895807 | 601767 | 4901083 |
| **CGCRI** | 101390 | 1159211 | 193175 | 2330283 |
| **CIMAP** | 278473 | 6773289 | 427459 | 11375818 |
| **CIMFR** | 41656 | 913265 | 83651 | 2250439 |
| **CLRI** | 439959 | 4964141 | 608885 | 7526688 |
| **CRRI** | 102831 | 679831 | 141531 | 1120318 |
| **CSIO** | 5065 | 33007 | 12360 | 80300 |
| **CSMCRI** | 99050 | 2484429 | 140055 | 4531498 |
| **NAL & 4PI** | 283665 | 2453768 | 540847 | 4394840 |
| **IGIB** | 22132 | 223607 | 92175 | 572530 |
| **IHBT** | 5501 | 206308 | 13996 | 283642 |
| **IICB** | 98459 | 1132196 | 190063 | 2285936 |
| **IICT** | 33402 | 162171 | 80751 | 624840 |
| **IIM** | 33776 | 888127 | 68614 | 1487962 |
| **IIP** | 10432 | 225618 | 28226 | 491505 |
| **IMMT** | 47455 | 669266 | 135130 | 2226436 |
| **IMTECH** | 5763 | 35288 | 12707 | 82586 |
| **NCL** | 747995 | 3939761 | 1339232 | 6565649 |
| **NEERI** | 468265 | 2418299 | 863062 | 4474971 |
| **NEIST** | 50446 | 1124683 | 121225 | 2436127 |
| **NGRI** | 130589 | 1483267 | 195609 | 3104542 |
| **NIIST** | 52200 | 1312992 | 131026 | 2384611 |
| **NIO** | 21173 | 213469 | 36670 | 265959 |
| **NISTADS** | 164071 | 1462845 | 260623 | 2756516 |
| **NPL** | 153367 | 1317108 | 230269 | 2309578 |
| **TKDL** | 148504 | 1242739 | 209748 | 2022700 |
| **HRDC** | 39724 | 1231939 | 140775 | 3501127 |
| **HQ** | 141531 | 1120318 | 188193 | 1744466 |
| **Pusa Comp.** | 177060 | 1607683 | 282752 | 3135388 |
| **Apartments** | 128389 | 908735 | 161466 | 1431094 |
| **NEERI,DEL** | 152580 | 1302120 | 225796 | 2246985 |
| **NEERI,HYD** | 111688 | 1082094 | 181627 | 2741836 |
