# Supplementary material for: Insights from a Pan India Sero-Epidemiological survey (Phenome-India Cohort) for SARS-CoV2
Source: eLife. 2021 Apr 20;10:e66537. doi: 10.7554/eLife.66537 (PMC8118652; doi:10.7554/eLife.66537)
Supplement: Figure 3—source data 2. [file elife-66537-fig3-data2.docx]

| **Male** | | |  | **Female** | | |
| --- | --- | --- | --- | --- | --- | --- |
| **n** | **Odds Ratio (95% CI)** | **P-Value** |  | **n** | **Odds Ratio (95% CI)** | **P-Value** |
| **Occupation: Odds of Being Sero-Positive for Outsourced Staff when compared to Regular Staff** | | | | | | |
| 6457 | **2.05 (1.73-2.42)** | <0.0001 |  | 2487 | **2.75 (1.95-3.89)** | <0.0001 |
| **Mode of Transport: Odds of Being Sero-Positive for Public Transport Users when compared to Private Transport Users** | | | | | | |
| 6375 | **1.91 (1.44-2.55)** | <0.0001 |  | 2398 | **1.83 (1.26-2.69)** | 0.01 |
| **Diet Type: Odds of Being Sero-Positive for Non-Vegetarian Subjects when compared to Vegetarian Subjects** | | | | | | |
| 6345 | **1.78 (1.45-2.19)** | <0.0001 |  | 2382 | **1.33 (0.97-1.82)** | 0.51 |
| **Smoking: Odds of Being Sero-Positive for Non-Smoking Subjects when compared to participants who Smoke** | | | | | | |
| 6379 | **1.62 (1.23-2.14)** | 0.0058 |  |  | Data Not Sufficient |  |
