# Supplementary material for: Insights from a Pan India Sero-Epidemiological survey (Phenome-India Cohort) for SARS-CoV2
Source: eLife. 2021 Apr 20;10:e66537. doi: 10.7554/eLife.66537 (PMC8118652; doi:10.7554/eLife.66537)
Supplement: Supplementary file 1. [file elife-66537-supp1.docx]

| **Symptom category** | **Gender** | |  |
| --- | --- | --- | --- |
|  | **Female** | **Male** | **Total** |
|  |  |  |  |
| **Asymptomatic** | 122 | 523 | 645 |
| **Symptomatic** | 76 | 138 | 214 |
| **Total** | 198 | 661 | 859 |
