# Supplementary material for: Insights from a Pan India Sero-Epidemiological survey (Phenome-India Cohort) for SARS-CoV2
Source: eLife. 2021 Apr 20;10:e66537. doi: 10.7554/eLife.66537 (PMC8118652; doi:10.7554/eLife.66537)
Supplement: Supplementary file 2. [file elife-66537-supp2.docx]

| **List of Symptoms** |  |  |  |
| --- | --- | --- | --- |
|  | **Count** | **Total Data** | **Rate** |
| **Fever** | 120 | 214 | 51.72414 |
| **Cough** | 81 | 214 | 34.91379 |
| **ST** | 64 | 214 | 27.58621 |
| **Anosmia** | 59 | 214 | 25.43103 |
| **Ageusia** | 54 | 214 | 23.27586 |
| **Tiredness** | 85 | 214 | 36.63793 |
| **Bodyache** | 57 | 214 | 24.56897 |
| **Headache** | 44 | 214 | 18.96552 |
| **Dyspnea** | 6 | 214 | 2.586207 |
