# Supplementary material for: Insights from a Pan India Sero-Epidemiological survey (Phenome-India Cohort) for SARS-CoV2
Source: eLife. 2021 Apr 20;10:e66537. doi: 10.7554/eLife.66537 (PMC8118652; doi:10.7554/eLife.66537)
Supplement: Supplementary file 3. — OR: odds ratio. [file elife-66537-supp3.docx]

| **Blood Group** | **Distribution in our Cohort** | **National Distribution** | **Positivity** | **Blood Group Vs Non-Blood Group Comparison within Cohort** | **P value**  **(Corrected)** | **Odds-Ratio**  **(OR)** |
| --- | --- | --- | --- | --- | --- | --- |
| **O** | 34.99 | 34.56 | 7.09 | O Vs Non O | 0.01 | 0.76 |
| **A** | 20.27 | 23.16 | 6.97 | A Vs Non A | 0.10 | 0.78 |
| **B** | 35.97 | 34.10 | 9.94 | B Vs Non B | 0.001 | 1.36 |
| **AB** | 8.76 | 8.18 | 10.19 | AB Vs Non AB | 0.35 | 1.27 |
